# Comparison of Two Surveys Using the Sexuality Assessment Tool (SexAT) in Flanders

**DOI:** 10.1093/geroni/igab046.658

**Published:** 2021-12-17

**Authors:** Els Messelis, Michael Bauer, Elisabeth Vander Stichele, els Elaut

**Affiliations:** 1 LACHESIS, Ghent, Oost-Vlaanderen, Belgium; 2 La Trobe University, Melbourn, Victoria, Australia; 3 AZ Sint Jan, Brugge, West-Vlaanderen, Belgium; 4 UGent, Gent, Oost-Vlaanderen, Belgium

## Abstract

From 2015 it is mandatory in Flanders, Belgium, to develop a policy to deal with sexual abuse in elderly care. Residential Aged Care Facilities (RACF'S) try to focus on this mandatory, but should also pay attention to implement an overall Sex and Intimacy Policy. This study contains a Comparison of two surveys (Messelis & Bauer, 2020 and Vander Stichele, e.a. 2020) in Flanders, Belgium, both using the Sexual Assessment Tool (SeAT, Messelis & Bauer, 2017). Both studies aimed to assess how supportive residential aged care facilities are of residents' sexual expression. In the survey of Messelis & Bauer 750 aged care facilities were contacted in 2017-2018 and 69 (9,2%) completed the SexAT survey after three reminders. Vander Stichele e.a. contacted 100 aged care facilities managers in 2019. Twenty of them (20% response rate) completed the SexAT after three reminders. Findings of the Messelis & Bauer survey indicate that 70% of the facilities rated 'very good' to 'good' (score between 21-59/69), while Vander Stichele e.a. found a prevalence of 76% of this score. Both found no facilities were rated 'excellent' (score greater than 60/69). In the category 'improvement needed' (score less than 20/69), percentages were 30% and 23%; a difference of 7% (CI95% of difference in percentage includes zero, not significant). There is room for improvement in residential aged care facilities for the support of sexual expression of residents. The more recent study confirms results of the previous one, and no significant evolution was observed in two consecutive cross-sectional surveys.

